# ‘The Listening Series’: increasing equity, diversity and inclusion in patient and public involvement and engagement for policy research by listening to and learning from under-represented groups

**DOI:** 10.1186/s40900-024-00601-2

**Published:** 2024-07-04

**Authors:** Charlotte Bevan, Fiona Alderdice, Sally Darby, Serena Gilzean-Hughes, Jenny McLeish, Sumayya Mulla, Rachel Plachcinski, Sophia Wilkinson, Harriet Williams, Rachel Rowe

**Affiliations:** 1 Patient and Public Involvement and Engagement (PPIE) co-lead, NIHR Policy Research Unit in Maternal and Neonatal Health and Care (PRU-MNHC), National Perinatal Epidemiology Unit, Nuffield Department of Population Health, University of Oxford, Old Road Campus, Oxford, OX3 7LF UK; 2 Co-Director, National Perinatal Epidemiology Unit, Nuffield Department of Population Health, NIHR PRU-MNHC, University of Oxford, Oxford, UK; 3 PPIE contributor, National Perinatal Epidemiology Unit, Department of Population Health, NIHR PRU-MNHC, University of Oxford, Oxford, Nuffield UK; 4 Researcher, National Perinatal Epidemiology Unit, Nuffield Department of Population Health, NIHR PRU-MNHC, University of Oxford, Oxford, UK; 5 Senior PPIE Officer, Nuffield Department of Population Health, University of Oxford, Oxford, UK

**Keywords:** Patient involvement, Patient engagement, Diversity, equity, inclusion

## Abstract

**Background:**

Policy research aims to provide evidence to inform government policy decisions about health and social care. Engaging and involving the public and patients in this work is widely recognised as essential. Research funders prioritise equality, diversity and inclusion (EDI) in patient and public involvement and engagement (PPIE), but people who are most likely to experience poor outcomes are also those least likely to be involved in research. This paper describes our experience of setting out to understand how to overcome barriers to EDI in PPIE in the research carried out by the National Institute for Health and Care Research (NIHR) Policy Research Unit in Maternal and Neonatal Health and Care (PRU-MNHC), in a PPIE consultation project we called *The Listening Series*.

**Methods:**

We convened five video-recorded online discussion groups involving 20 individuals advocating for groups who are under-represented in our research. Those taking part included people working with Black and Asian women and families, young parents, those from socially deprived backgrounds, and women and families with physical and learning disabilities. Discussions focussed on practical solutions to addressing challenges to people being excluded, and how to improve EDI in our research.

**Learning and reflection:**

Five key themes were identified: ‘build trust’; ‘involve us from the beginning’; ‘show us impact’; ‘use clear, appropriate and inclusive communication’; and ‘imagine life in our shoes’. We used the learning to create a guidance document for researchers and an accompanying 15-minute film. We also took practical steps to embed the learning strategically by expanding our Task Group for PPIE in the PRU-MNHC to include four *Listening Series* invitees with a remit to champion EDI in our research and ensure that it is embedded in our PPIE activities. We continue to reflect on and work to address the associated challenges.

**Conclusions:**

*The Listening Series* helped us rethink our processes for inclusion to go beyond traditional methods of involvement and engagement. The themes identified pose challenges that require time, resource and empathic engagement from researchers to be meaningfully resolved. This has implications for policy makers and research funders who need to consider this in their processes.

## Abstract (plain language summary)

**What we know:**

It is important that health care researchers involve patients and the public from a wide range of social and ethnic backgrounds in research, but we know that this often does not happen. We are a group of researchers and patient/public representatives, working in research to improve care for pregnant women and babies. We wanted to find out how to involve people from more diverse backgrounds in our research.

**What we did:**

We organised five online discussion groups with 20 people working with Black and Asian families, young parents, those from socially deprived backgrounds and parents with physical or learning disabilities. We asked them what we should do to involve a wider range of people in our research. We called this *The Listening Series*. We summarised the most important things people said in a written guide for researchers and a short film. We then asked people who had been invited to take part in *The Listening Series* to join us to develop new ways of working together.

**What we learned:**

The five themes we identified were: ‘build trust’; ‘involve us from the beginning’; ‘show us impact’; ‘use clear, appropriate and inclusive communication’; and ‘imagine life in our shoes’. In summary, researchers need to take the time to build trusting relationships with patients and the public; actively listening and learning from them. This can be challenging for researchers and patient representatives. Research funders need to allow time and money for this to happen in a meaningful way.

## Background

Policy research aims to provide evidence to inform government policy decisions about health and social care [1]. In England, the Department of Health and Social Care, through the National Institute for Health and Care Research (NIHR), funds a number of Policy Research Units (PRU) to undertake this research. This includes the NIHR Policy Research Unit in Maternal and Neonatal Health and Care (PRU-MNHC), where most of the authors of this paper are based, which specialises in research to improve health and care for pregnant women, babies and families [2]. Engaging and involving the public and patients in this work helps ensure that the research is informed by, as well as relevant and accessible to those most impacted by its findings. A more ‘rights-based’ framing of involvement would say that people who are most affected by the outcomes of publicly funded policy research, have the right to be involved in it [3].

There are stark social, socio-economic, demographic and geographical inequalities in many health outcomes [4]. In maternity care, women from South Asian and Black ethnic groups and those living in the most deprived areas are more likely to have pregnancy complications, such as hypertension or diabetes, and they and their babies are at increased risk of dying during pregnancy or after birth [5–7]. Research funders prioritise equality and inclusion in patient and public involvement and engagement (PPIE) [8], but people who are most likely to experience poor outcomes are also those least likely to take part in conversations about research, attend research-related events, join advisory groups or enrol as research participants [9–11].

The barriers to involvement are well-documented. These may be practical and organisational, such as travel, transport, timing and location of meetings; personal (physical and/or emotional); relational or cultural, including pre-existing beliefs or expectations on the part of researchers and others; or related to language, including not speaking English as a first language, but also the technical language and jargon often used by researchers [9, 12, 13]. People experiencing these barriers have been described as ‘hard to reach’ [14], suggesting the agency for being ‘unreachable’ rests with the individuals themselves. Redefining people as ‘seldom listened to’ or under-represented helps re-orientate the responsibility onto the research community and means that researchers must seek to find solutions to these barriers, and new ways to engage listen more actively and effectively [15].

In the PRU-MNHC we have sought to involve a broad range of third sector representatives in our research, but in the past have worked most actively with representatives of well-established charities. Some individual PRU-MNHC researchers have developed sustained relationships with individual PPIE contributors representing more diverse communities, often for specific research projects, but these individuals and groups have not been as actively involved in our broader programme of work. We were keen to improve this but recognised that we had to listen to those we wanted to involve, to learn from them how best to achieve this.

This paper describes our experience of setting out to understand how to overcome barriers to equity, diversity and inclusion (EDI) in PPIE in the research carried out by the NIHR PRU-MNHC, in a PPIE consultation exercise we called *The Listening Series*. We wanted to consult more widely among diverse groups of maternity service users to help us understand how to be more inclusive in our PPIE. We sought to achieve this by:

- Listening to, and learning from, people who are under-represented in our research, through virtual group discussions.
- Developing resources for researchers to raise awareness of EDI and support them to improve their PPIE.

While the focus of *The Listening Series* was EDI in PPIE for maternity and neonatal care research, the principles are transferrable to other study fields. We share our experiences of carrying out this consultation, and our findings, to support those seeking to improve EDI in PPIE in their own areas of research. We also reflect on our experiences of embedding this learning in our processes and some of our further learning and reflections as a result.

## The Listening Series

### Identifying representatives of ‘under-represented’ groups

We used a range of methods to identify people to take part in this consultation. We reviewed our list of existing PPIE stakeholders (comprising around 60 organisations and individuals at that time) to identify those advocating for under-represented groups. These stakeholders received PRU-MNHC communications, but not all were actively engaged or involved in our research, and most had never attended our annual stakeholder days, where we consulted about women’s and families’ priorities for our research. We also read reports authored by charities highlighting maternity inequalities to identify other advocates working in this area. Using these approaches we identified 24 organisations and individuals representing and working with under-represented groups, with whom the PRU-MNHC did not already have a close working relationship.

### Planning and running the consultation

We sought advice about how best to generate open conversations from a mentor and coach with 30 years’ experience facilitating workshops with clients, including large corporations, universities and charities. Following this we decided to convene small online discussion groups, facilitated by our PPIE co-leads.

We invited 24 people to attend one of five discussion groups in June 2021; all accepted the invitation, but not all were able to attend one of the five dates offered. In total, 20 people working with a wide range of social and ethnic communities attended, including Black and Asian women and families; young parents; parents from socially deprived backgrounds; and women with physical or learning disabilities. Each group comprised three to five participants; our two PPIE co-leads as facilitators; and a senior PRU-MNHC researcher who attended to help clarify any questions about PRU-MNHC research.

Before each group, participants were sent some pre-reading, giving examples of the research carried out by the PRU-MNHC, and inviting them to consider ways of overcoming barriers to involvement and engagement. Discussions focussed on practical solutions to addressing challenges to people feeling and being excluded, and how to improve EDI in our research. Discussions lasted around two hours and were video-recorded. None of the attendees needed language support, but we supported a partially-sighted attendee by describing visual content on the day. Participants were paid for their time spent on preparation and attendance, using NIHR rates for public involvement in research [16].

### Identifying key themes

The PPIE co-leads made a transcript of the recordings and identified key themes by noting recurring words, phrases and topics, and grouping and categorising these, through iterative discussion with each other, and with those who attended.

### What participants said

We identified five key themes from *The Listening Series*, which need to be addressed at a strategic level as well as within individual projects. The themes were:

#### Build trust

Participants said that trust was at the heart of all good public involvement, indicating that this could be built through ‘trusted intermediaries’ – that is organisations who already represent families and who might act as relationship brokers between families with lived experience and researchers. This type of relationship-building had to be sustained, however, through further principles around being seen as equal partners and strategic allies, not just as people who provide their lived experiences as part of research, for free, with no obvious positive outcome. They said this was particularly important for people from groups who have mistrust in institutions because of the way they have been treated, either historically or currently as an individual.

*“There’s a real need to build that trusting relationship from the start. I know from conversations with different ethnic groups of parents, there’s a lot of mistrust on where the data’s going and what’s going on with it. They don’t feel they’ve been listened to in their pregnancy so why would they be listened to now?”* (Chair of charity providing support for bereaved families and promoting awareness of cultural differences in pregnancy loss and the death of a baby around the time of birth).

#### Involve us from the beginning

Building on trust, participants said that involving people from the start of a project is important because it represents more genuine involvement, where their input is valued at every stage. One contributor, from a charity working with mothers and babies facing inequalities and disadvantage, said that bringing people with ‘lived experience’ of a condition to research discussions early on should be *“a first thought and not a middle or last thought”*. Others said that sometimes people could feel *”exploited”*, that they were only being *”brought out”* when it was convenient to the researchers, often when the study results needed to be disseminated.

*“Sometimes you guys have a project in mind, you know how you want it to go, and it’s very rigid, not much fluidity. Having those honest conversations at the very beginning and shaping it based on that is very important.”* (Chief Executive Officer of an organisation supporting Black mothers).

#### Show us impact

Participants said that people may not have much time, but they do want improvements in care. It is therefore key to show them the impact that sharing their experiences and their knowledge had on the research, and ultimately on health policy. This was captured by the phrase: ‘You said, we did’. Participants said that it was critical to know how research has directly made a difference to their communities.

*“I really like the ‘you said, we did’… that feedback loop… If you know someone is listening to you, you will speak more, you will have that confidence.”* (Founder of a peer-support group in the North of England for South Asian mothers).

*“What would motivate Black mums to take part? You’d have to try really hard… Whenever data comes out about us it’s never favourable, it doesn’t paint us in a good light or show our experiences are good. So we’re a bit tired of it to be honest, and unless we can see that something concrete is going to come out of this, and not just another load of statistics then what’s the point.”* (Co-founder of a London-based collective raising awareness around the experiences of Black mothers).

#### Use clear, appropriate, and inclusive communication

Participants who advocate for families with a low reading age, or who don’t speak English as a first language, said that these language issues act as barriers to engaging with research. One participant who represents mothers living with disability told us that access to information, and invitations that were ‘personalised’ were important:

*“Access is absolutely a barrier for people with disabilities. A lot wouldn’t feel the research was for them, but for the parenting mainstream. There needs to be an explicit invitation to make them feel they are wanted and included.”* (Founder of an organisation supporting mothers with physical disabilities).

As a minimum, researchers should use straightforward jargon-free language, but they should also be flexible about approaches to communicating with people, ask people what works best for them, using platforms and formats to suit people’s diverse needs.

#### Imagine life in our shoes

Participants cautioned that if someone is trying to find a home, a job or look after a young family, taking part in research will not be a priority for them, particularly if it doesn’t feel relevant to their daily life. They said that imagining the lives of the audience you are hoping to involve will help target an approach to engaging them.

*“I think that some disadvantaged communities… are sort of over-burdened by the difficulties in their lives and so the bits that we’ve already said about reaching out… about going to those places that those families hopefully will frequent are the ways to engage them. We can’t assume parents will have the motivation and the space to think ‘Oh if I got involved in research things will change’.”* (Director of an organisation working to support parents and families affected by neglect, domestic violence, mental illness and substance use).

## The resources we developed

We used the learning from *The Listening Series* to create a guidance document for researchers [17] and an accompanying 15-minute film using soundbites from the discussions [18]. During development, we shared the guidance and film with participants to ensure it accurately reflected their views and perspectives, and with researchers to make sure these resources met their needs.

## Participant experiences

We sent a short survey to participants immediately after each group, with feedback from the first groups shaping the content, length, and facilitation of later groups. In particular, participants asked for clearer information about the research methodologies being discussed, longer meetings to allow more time for discussion, and more active facilitation to ensure that all voices were heard. Seventeen out of 20 participants responded. Fourteen strongly agreed that they had enjoyed taking part in their *Listening Series* discussions and all said they felt listened to.

## Reflections on the process

Using online meetings, inviting representatives of organisations or advocates for particular communities, meant we were able to hear the perspectives of a broad range of people from under-represented groups, reaching them directly in their homes (some had young children) and offices. Keeping the groups small and informal and allowing sufficient time meant participants had plenty of opportunity to speak. The groups rarely ran out of things to say and after the first two meetings we extended subsequent meetings by 15 min. By creating a platform of small group online meetings that were respectfully facilitated, guided towards specific goals, and where participants felt genuinely listened to, we were able to open up discussions that were both honest and positive, which led to learning and fostered more trusted relationships.

We originally intended to make audio podcasts from *The Listening Series*, but the participants spoke so engagingly that we decided a film would more vividly represent what participants said, would have a greater impact on researchers and was likely to be shared more widely. We underestimated the time and resources that making a film would take, and were fortunate that our team included people with broadcast journalism experience and that we had support from an in-house graphic designer.

We carried out an initial evaluation of researchers’ perceptions of our outputs, with positive responses, but numbers were small so this was not a robust assessment of impact and is therefore not reported here.

## Embedding learning

In June 2022, we invited people we had originally asked to join us for *The Listening Series* to a face-to-face meeting to explore ‘what next?’, including how we might embed the five themes in the work of the PRU-MNHC. All agreed that inclusion should take place at a strategic level and not just for individual studies. The result was that four attendees at this meeting (SM, SGH, SD, HW) (three who took part in *The Listening Series* and a further invitee, who had been unable to attend the original discussion groups) joined our ongoing Task Group for PPIE, with a specific remit to ‘champion’ the involvement of under-represented groups in PRU-MNHC research. These new Task Group members are supported by regular meetings, mentoring and training from our PPIE co-leads and are paid for their time, using NIHR rates for public involvement in research. In the PRU-MNHC funded for 2024-8 this group is supporting active monitoring of EDI in our PPIE.

## Learning and reflections since

Improving EDI in PPIE is essential for research, but addressing this can only be achieved by consulting and learning from those who are seldom listened to. *The Listening Series* helped us rethink our processes for inclusion to go beyond traditional methods of engagement and embed representatives more strategically as ‘equal partners’. In our Task Group (which includes nine of the ten co-authors of this paper), we continue to reflect on and discuss this work and we share some of these reflections and our experiences here.

### The challenges around building trust

Many of those from under-represented communities who get involved in research may be doing so for the first time and may be starting from a point of substantial mistrust. In the PRU-MNHC we had built strong trusting relationships with many PPI contributors over the years. *The Listening Series* however, and our work together since, has highlighted how trust is particularly salient for those from under-represented groups. We still sometimes misunderstand each other’s motives, and have had some challenging conversations as a result. Payment for PPI, with differing perceptions around the relative value and meaning of vouchers versus cash payments, and delays caused by cumbersome university payment systems, have often brought mistrust to the fore. For us *The Listening Series* highlighted the importance of trust, and helped us to start building it, but trust is easily lost, and clear communication, honesty and openness are key to sustaining it.

### The need for additional training, resources and support

The idea of training, support and mentoring for new PPI contributors is not new [19]. It is also not specific to PPI contributors from under-represented communities, but may be more likely to be needed given historic lack of involvement. Our PPIE co-leads, and more recently a paid PPI facilitator, have helped build relationships and trust, identify individual needs, and devise appropriate training and support. This has included bespoke sessions on understanding other perspectives, using plain language, and recording and reporting PPI, with informal mentoring through an online chat group.

### Challenges around building reciprocity

Financial rewards are not the sole goal of organisations. We had started to consider the principle of reciprocity in our PPI, but this was further reinforced by individuals in *The Listening Series*. Addressing this not only means involving groups from the beginning of any project, but also means giving back by supporting communities’ own goals, which may not be linked directly to the work that researchers are focussed on delivering. In the PRU-MNHC we are exploring innovative ways of ‘giving back’ to local community organisations working in our field, so that these relationships become more genuinely reciprocal and less burdensome to them.

### The need for ongoing engagement with research funders

As researchers and research funders become increasingly aware of the need to consider EDI in PPIE [8], some organisations have told us that they have been overwhelmed with requests to support research projects. Many small, and even larger, community organisations do not have the resources to support multiple requests for involvement in research. For some ‘stretched’ PPI representatives routinely called upon to represent their community, NIHR payments are increasingly not seen as sufficient recompense, when their primary role is to delivery front-line services and/or wider engagement activities.

In our PPIE Task Group we have observed that there is also a risk that once a single representative from an under-represented group has been engaged, funders and researchers imagine that a box has been ticked. Not only does this put pressure on an individual or organisation to continually speak for an entire group, but it also risks continuing a cycle of engaging the ‘usual suspects’ without reflection on who may still be missing.

Ultimately research funders should ensure that they allow for the time and resources required to consider EDI in a meaningful way for PPIE when they develop calls for research proposals and assess research applications. This will help ensure that researchers themselves are not forced into the corner of making EDI simply a box-ticking exercise, ultimately undoing the trust they may have built with their PPIE partners.

## Conclusions

In summary, our experience is that involving a wide range of people in our research is an on-going endeavour that is unlikely to be addressed by a single toolkit or process. As we have seen ourselves in our attempts to improve EDI, we are likely to, at best, keep getting it ‘a little bit wrong’ and, at worst, keep missing a key part of the jigsaw. Iterative consultation and learning, therefore, with those most likely to be left out of research, will be the key to making EDI improvements genuinely meaningful and reflective of society’s dynamically changing needs.

## Data Availability

No datasets were generated or analysed during the current study.

## References

[1] National Institute for Health and Care Research. Policy Research London: NIHR. 2023 [Accessed 14th December 2023]. https://www.nihr.ac.uk/explore-nihr/funding-programmes/policy-research.htm.

[2] National Perinatal Epidemiology Unit. PRU-MNHC Oxford: National Perinatal Epidemiology Unit, University of Oxford. 2023 [Accessed 14th December 2023]. https://www.npeu.ox.ac.uk/pru-mnhc.

[3] Russell J, Fudge N, Greenhalgh T (2020). The impact of public involvement in health research: what are we measuring? Why are we measuring it? Should we stop measuring it?. Res Involv Engagem.

[4] Watt T, Raymond A, Rachet-Jacquet L. Quantifying health inequalities in England London: The Health Foundation; 2022 [Accessed 14th December 2023]. https://www.health.org.uk/news-and-comment/charts-and-infographics/quantifying-health-inequalities.

[5] Webster K, NMPA Project Team. Ethnic and Socio-economic Inequalities in NHS Maternity and Perinatal Care for Women and their Babies. and 31 March 2018 across England, Scotland and Wales. London: RCOG; 2021. : Assessing care using data from births between 1 April 2015.

[6] Draper E, Gallimore I, Smith L, Matthews R, Fenton A, Kurinczuk J et al. MBRRACE-UK Perinatal Mortality Surveillance Report, UK Perinatal Deaths for Births from January to December 2020. Leicester: The Infant Mortality and Morbidity Studies, Department of Health Sciences, University of Leicester; 2022.

[7] Knight M, Bunch K, Patel R, Shakespeare J, Kotnis R, Kenyon S (2022). Saving lives, improving mothers’ Care Core Report - lessons learned to inform maternity care from the UK and Ireland Confidential enquiries into maternal deaths and morbidity 2018-20.

[8] Imison C, Kaur M, Dawson S. Supporting equity and tackling inequality: how can NIHR promote inclusion in public partnerships? An agenda for action. London: National Institute for Health and Care Research; 2022. https://www.learningforinvolvement.org.uk/content/resource/supporting-equity-and-tackling-inequality-how-can-nihr-promote-inclusion-in-public-partnerships.

[9] Beresford P. Beyond the Usual Suspects. London: Shaping our Lives; 2013 8th September 2023]. https://shapingourlives.org.uk/report/beyond-the-usual-suspects-research-report/.

[10] National Institute for Health and Care Research. NIHR Public Involvement Feedback Survey 2020–2021: The results London: NIHR. 2022 [Accessed 8th September 2023]. https://www.nihr.ac.uk/documents/nihr-public-involvement-feedback-survey-2020-2021-the-results/29751.

[11] Bower P, Grigoroglou C, Anselmi L, Kontopantelis E, Sutton M, Ashworth M (2020). Is health research undertaken where the burden of disease is greatest? Observational study of geographical inequalities in recruitment to research in England 2013–2018. BMC Med.

[12] healthtalk.org. Patient and public involvement in research: Difficulties and barriers to involvement Oxford: The Dipex Charity. 2019 [Accessed 8th September 2023]. https://healthtalk.org/experiences/patient-and-public-involvement-research/difficulties-and-barriers-to-involvement/.

[13] Ocloo J, Garfield S, Franklin BD, Dawson S (2021). Exploring the theory, barriers and enablers for patient and public involvement across health, social care and patient safety: a systematic review of reviews. Health Res Policy Syst.

[14] Flanagan SM, Hancock B (2010). Reaching the hard to reach’--lessons learned from the VCS (voluntary and community Sector). A qualitative study. BMC Health Serv Res.

[15] Islam S, Joseph O, Chaudry A, Forde D, Keane A, Wilson C (2021). We are not hard to reach, but we may find it hard to trust … Involving and engaging ‘seldom listened to’ community voices in clinical translational health research: a social innovation approach. Res Involv Engagem.

[16] National Institute for Health and Care Research. Payment guidance for researchers and professionals London: NIHR. 2022 [Accessed 14th December 2023]. https://www.nihr.ac.uk/documents/payment-guidance-for-researchers-and-professionals/27392.

[17] Plachcinski R, Alderdice F, Bevan C, Wilkinson S, Rowe R. The Listening Series: including everyone in public engagement with research. Guidance for researchers. 2022 Date Accessed. https://tv.ndph.ox.ac.uk/wp-content/uploads/2022/01/Listening-Series-Guide-for-Researchers.pdf.

[18] Policy Research Unit in Maternal and Neonatal Health and Care. Listening Series [Video]. Oxford: Oxford Population Health. 2022 [ https://tv.ndph.ox.ac.uk/ppie/.

[19] INVOLVE (2012). Developing training and support for public involvement in research.

